# Improved spectral resolution of the femtosecond stimulated Raman spectroscopy achieved by the use of the 2nd-order diffraction method

**DOI:** 10.1038/s41598-021-83090-7

**Published:** 2021-02-09

**Authors:** Dong-gu Kang, Kyung Chul Woo, Do Hyung Kang, Chanho Park, Sang Kyu Kim

**Affiliations:** 1 Department of Chemistry, KAIST, Daejeon, 34141 Republic of Korea; 2 Division of Chemistry and Biological Chemistry, School of Physical and Mathematical Sciences, Nanyang Technological University, 21 Nanyang Link, Singapore, 637371 Singapore

**Keywords:** Applied optics, Optical techniques

## Abstract

Prolongation of the picosecond Raman pump laser pulse in the femtosecond stimulated Raman spectroscopy (FSRS) setup is essential for achieving the high spectral resolution of the time-resolved vibrational Raman spectra. In this work, the 2nd-order diffraction has been firstly employed in the double-pass grating filter technique for realizing the FSRS setup with the sub-5 cm^−1^ spectral resolution. It has been experimentally demonstrated that our new FSRS setup gives rise to a highly-resolved Raman spectrum of the excited *trans*-stilbene, which is much improved from those reported in the literatures. The spectral resolution of the present FSRS system has been estimated to be the lowest value ever reported to date, giving Δν = 2.5 cm^−1^.

## Introduction

Femtosecond stimulated Raman spectroscopy (FSRS)^1,2^ is one of the most versatile time-resolved vibrational spectroscopic tools in tracking the nuclear motions on the excited states through Stokes and/or anti-Stokes Raman signals with the sub-picosecond (ps) temporal resolution. FSRS has been extremely successful in interrogating a number of chemical and biological processes in the systems of organic/inorganic compounds^3,4^, biomolecules^5,6^, polymers^7,8^, or nanocrystals^9,10^. The FSRS setup consists of three different laser pulses. The femtosecond (fs) actinic pulse is used to generate the wavepacket on the excited states, which is followed by the pair of the narrow-bandwidth ps-pump and broad-bandwidth fs-probe laser pulses for the stimulated-Raman (SR) process. Time-resolved Stokes or anti-Stokes signals may access the real-time evolution of the vibrational structure of the excited-state. As the SR signal monitored as a function of the delay time between the actinic and Raman probe laser pulses is often quite weak, the proper wavelength tuning of the Raman pump laser pulse is essential to obtain the high-quality spectra. Furthermore, the extension of the temporal duration of the Raman pump is critical in getting the high spectral resolution^11,12^.

In many laboratories, the ps Raman-pump laser pulse is generated by the optical conversion from the fundamental fs laser pulse. For instance, in the second-harmonic bandwidth-compressor (SH-BC) technique, the sum frequency generation of two split fs fundamental pulses having opposite chirps is utilized to generate the ps laser pulse output with the Fourier-transform limited bandwidth of 5–10 cm^−1^^13,14^. Although the SH-BC has the merits of the high conversion efficiency and the narrow spectral bandwidth, the wavelength tunability is quite limited as well as the optical setup is quite complicated. Accordingly, the diverse advanced techniques including the optical parametric amplifier (OPA) system with double pass grating filter (DPGF) method have been developed to extend the wavelength tunability of the ps Raman-pump pulse to cover the ultraviolet to near-infrared region despite the spectral broadening (8–30 cm^−1^)^15–21^. Utilizing the group velocity mismatch in the second-harmonic generation (SHG), the spectral compression could be achieved in the DPGF method to produce the ultraviolet or visible ps laser pulse with the bandwidth less than 10 cm^−1^^22,23^. In tailoring the appropriate ps laser pulse for FSRS, the DPGF technique seems to be essential despite its disadvantage of the energy loss.

The DPGF setup simply consists of a grating, a lens and an adjustable slit. In the frequency domain, the grating disperses the laser pulse into the individual optical components whereas the lens spatially separates them into diffraction-limited points in the Fourier plane. The 1st-order diffraction of grating is widely employed in this stage. By adjusting the slit width near the focal point, the spectral bandwidth is narrowed down although the significant loss of the laser intensity occurs at the same time. The laser pulse through the slit then refocuses on the grating to produce the final output ps laser pulse which has been minimized with respect to both the angular dispersion and spatial chirp^24,25^. In principle, as the higher order diffraction of the grating is generally much more effective in the spectral dispersion, the DPGF technique with the 2nd-order diffraction, compared to the 1st-order diffraction, is expected to give the ps laser output with a much improved spectral resolution. Surprisingly, probably due to the overestimated conjecture of the gigantic power loss in the high-order diffraction, the DPGF technique employing the 2nd-order diffraction has never been demonstrated to date. Here, we have firstly employed the 2nd-order diffraction DPGF technique in the actual FSRS setup. At various circumstances, we have compared the detailed performances of the 1st- and 2nd-order diffraction methods. The FSR spectrum of the excited *trans*-stilbene has been obtained for the demonstration of the new technique in terms of the spectral resolution of the time-resolved Raman spectrum.

## Experimental setup

**Figure 1:**
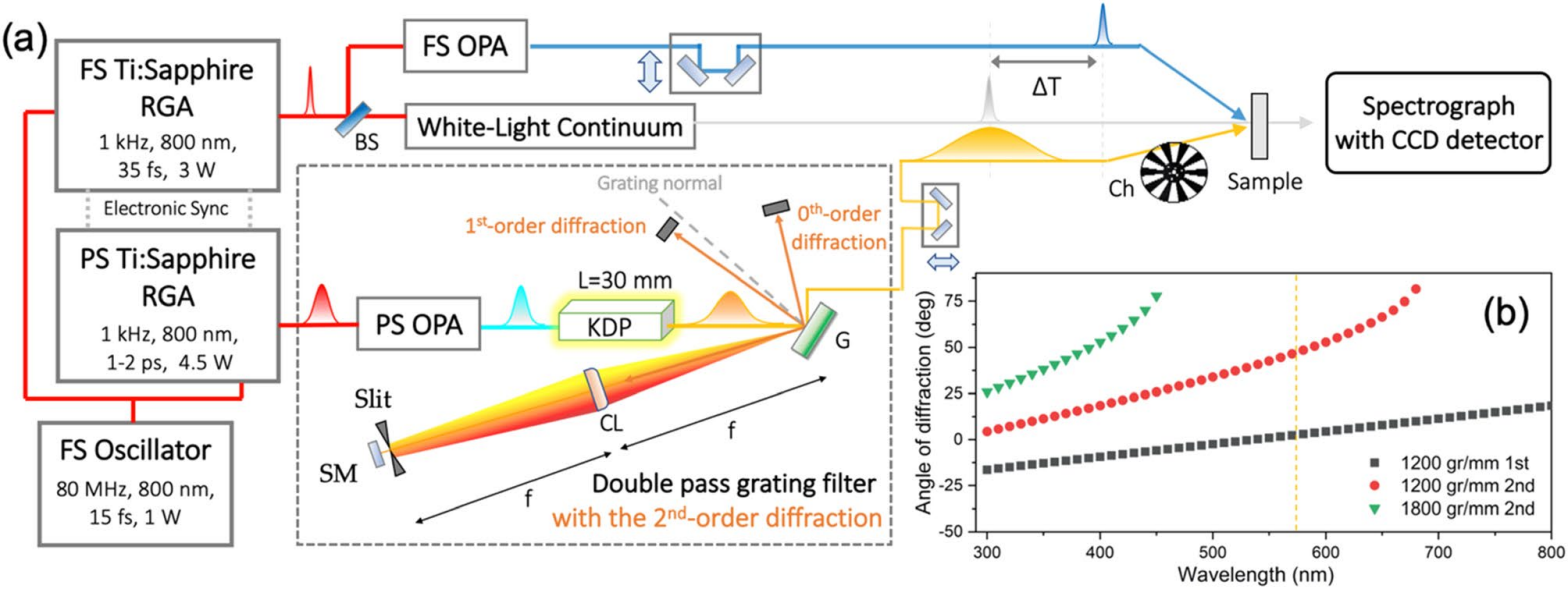
(**a**) The schematic of the synchronized fs and ps Ti:Sapphire RGA laser systems and the optical layout for FSRS using the DPGF employing the 2nd-order diffraction. The laser output from the fs RGA generates the wavelength-tunable actinic pump pulse (blue line) and the white-light continuum Raman-probe pulse (gray line). The DPGF with the 2nd-order diffraction tailors a narrow bandwidth ps Raman-pump pulse (yellow line). All three pulses are focused on the sample while the Raman-probe pulse was detected by the spectrograph coupling CCD detector. *BS* beam splitter, *G* grating, *CL* cylindrical lens, *SM* silver mirror, *Ch* optical chopper. (**b**) Angle of the 1st- or 2nd-order diffraction of the grating is shown as a function of the wavelength of input beam with the incidence angle of 40°. The yellow dash line is the wavelength of 575 nm using Raman-pump pulse.

The layout of the FSRS setup equipped with the dual fs/ps Ti:Sapphire regenerative amplified (RGA) laser systems is shown in Fig. 1. The fs laser pulse from a single oscillator (Vitara-T HP, Coherent) was divided in two, and each one was seeded into the fs-RGA (Legend Elite-USP, Coherent, 1 kHz) or the ps-RGA (Legend Elite-Pico, Coherent, 1 kHz) system. The delay generator (SDG Elite, Coherent) of the pump lasers (Evolution/Revolution, Coherent) in two RGA systems were finely controlled by the single master delay generator for the synchronization of two outputs. The resultant synchronized fs/ps laser pulses were then further controlled by the linear translational stages.

The tailoring process of the ps Raman-pump pulse consisted of two steps. First, the ps near-infrared (NIR) laser pulse (*λ* ~ 1150 nm, 380 μJ/pulse) from the ps-OPA (TOPAS-800, Light Conversion) was passed through a 30 mm long KDP crystal (CASTECH, θ = 50.5°, Ф = 45°) to generate the SHG output. Its conversion efficiency is estimated to be 25% (*λ* ~ 575 nm, 95 μJ/pulse). Since the group-velocity mismatch between the NIR input and SHG output pulses was so huge in the nonlinear mixing crystal, the bandwidth of the ps output laser pulse was already narrowed down significantly. Though the refractive index of 25 mm BBO crystal is larger than that of KDP crystal, we have chosen the latter to reduce the unwanted artifacts caused by super-fluorescence and/or saturation^22,26^. Secondly, the output from the KDP enters the DPGF scheme where a grating of 1200 grooves/mm was used. The diffraction equation for the light at the wavelength of *λ* is given as follows.

$$ m\lambda = d\left( {\sin \theta_{i} + \sin \theta_{m} } \right) $$

Here, *m* is the diffraction order, *d* is the spacing between adjacent grooves, *θ*_*i*_ is the incidence angle, and *θ*_*m*_ is the diffraction angle. It is notable that the 2nd-order diffraction from the 1200 grooves (gr)/mm grating is quite appropriate for dispersing the laser pulse in the wide wavelength range, Fig. 1b. The 2nd-order diffraction from the 1800 gr/mm grating could not be employed as the Raman pump at 575 nm due to the large diffraction angle. The optical path length from the plane ruled grating (20RG1200-1000-2, 1200 gr/mm 1000 nm blaze, Newport) to a cylindrical lens (f.l. = 500 mm, LJ1144L2-A, Thorlabs) was adjusted to be identical to that from the latter to a silver mirror, Fig. 1. The Fourier plane was generated to give the chirp-free laser pulse whereas the adjustable slit (VA100C/M, Thorlabs) was used to reduce the spectral width of the final output (at ~ 575 nm, ~ 2.2 μJ/pulse). The broad-band fs Raman-probe pulse was obtained from the white-light continuum (WLC) generation by focusing the fundamental fs pulse to a 2 mm-thick sapphire plate (NewLight), giving the cross-correlation width of ~ 80 fs when it is combined with the actinic fs-pump pulse (see Supplementary Information). Then, it interacts with the ps Raman-pump pulse at the sample to give the stimulated Raman signal which was detected by the spectrograph (SP-2358, Princeton Instruments) equipped with the charge coupled device (PIXIS-100BR excelon, Princeton Instruments).

## Results and discussion

**Figure 2:**
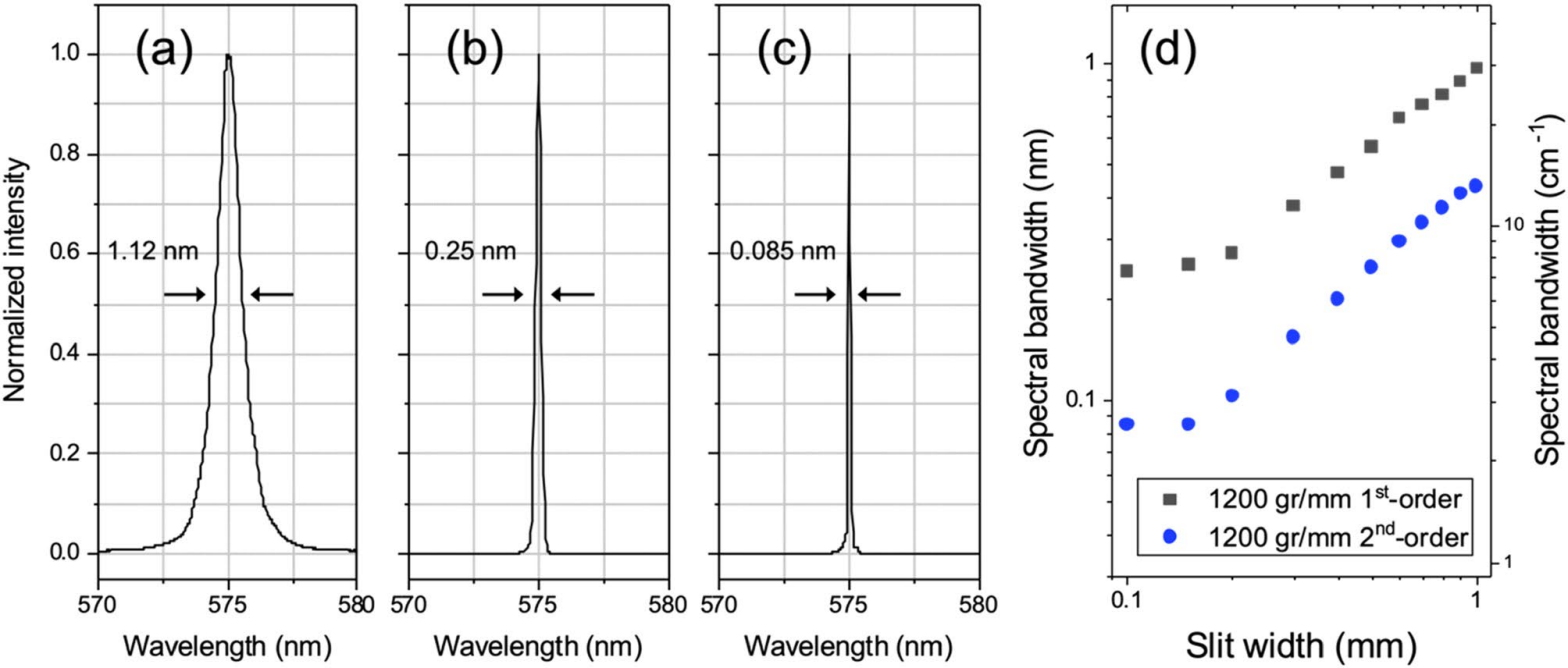
Normalized spectra of Raman-pump pulse for (**a**) the bare output from the ps-OPA, the SHG output from the (**b**) 1st- and (**c**) 2nd-order diffraction DPGF methods at the 0.15 mm slit width. (**d**) Spectral bandwidth fitted by Voigt function depends on the slit width in DPGF using the 1st- and 2nd-order diffraction. The 1800 gr/mm grating (500 nm blaze) was used in the spectrometer.

**Figure 3:**
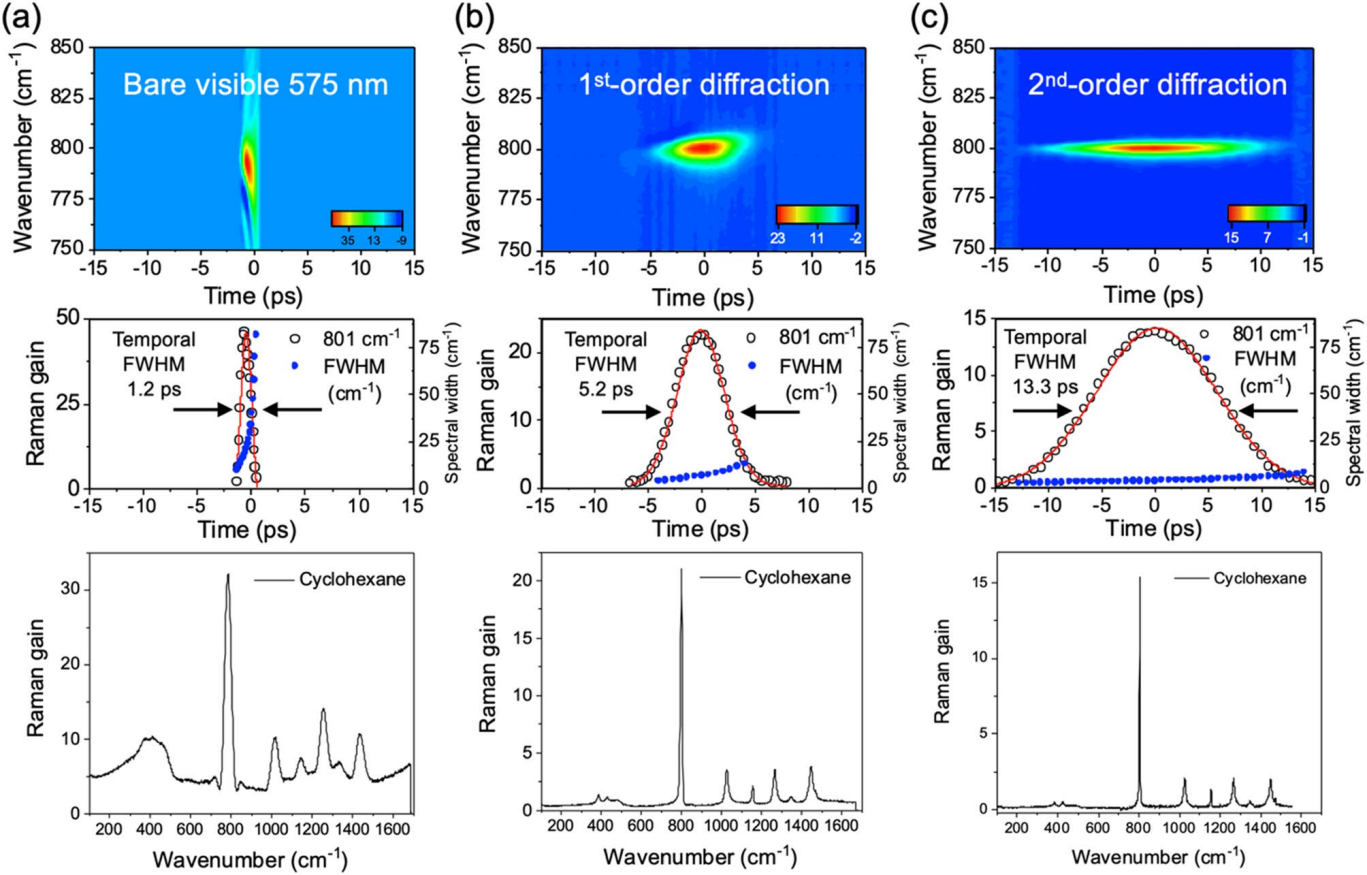
(Top) Two dimensional cross-correlation measurements of the stimulated Raman band at 801 cm^−1^ of cyclohexane using the 575 nm ps Raman-pump pulse and the fs broad Stokes Raman-probe pulse. Three different Raman-pump pulse of (**a**) the bare output from the ps-OPA, (**b**) the SHG output from the 1st-order diffraction DPGF, and (**c**) the SHG output from the 2nd-order diffraction DPGF are shown. The power of Raman-pump pulse was ~ 700 nJ/pulse in all three experimental condition. (Middle) Temporal cross-correlated FWHMs (the opened black circle) of the 801 cm^−1^ Raman gain of cyclohexane at the slit width of 0.15 mm. Spectral FWHMs (the filled blue circle) of the 801 cm^−1^ Raman band as a function of the delay-time. (Bottom) The whole stimulated Raman spectra of cyclohexane has been obtained at the delay time between the Raman-pump and -probe pulses which is optimized for the maximum Raman gain. For the stimulated Raman signal, the 1200 gr/mm grating (1000 nm blaze) was employed in the spectrometer for utilizing the more dispersive nature of the grating.

In order to characterize the tailored Raman-pump pulses from the DPGF setup at various experimental conditions, their spectral profiles as well as integrated intensities are measured as a function of the slit width for (i) the direct ps-OPA output, the SHG output from (ii) the first-order, or (iii) second-order diffraction DPGF scheme, Fig. 2. The full width at the half-maximum (FWHM) of the corresponding Raman pulse shows the sharp decrease from (i) 1.12 nm to (ii) 0.25 or (iii) 0.085 nm at the slit width of 0.15 mm for the last two cases. In order to compare temporal widths of the Raman-pump pulses generated in different conditions, the stimulated Raman signals of cyclohexane have been obtained as a function of the delay time between the ps Raman pump and fs broadband Stokes probe laser pulses, Fig. 3. Cross-correlation band widths of the stimulated Raman band at 801 cm^−1^ of cyclohexane are obtained with the Raman-pump laser pulse in the (i), (ii) or (iii) conditions. The CC stretching vibrational mode (A_1g_) at 801 cm^−1^ of cyclohexane is chosen as it is ideal for the spectral resolution measurement^1^. FWHM of the cross-correlation Raman signal is found to be 1.2, 5.2 or 13.3 ps for (i), (ii) or (iii), respectively. Accordingly, the spectral width of the Raman band is found to be narrowest in the (iii) condition (vide infra). It should be noted that the Raman spectrum obtained with the bare output from the ps-OPA (i) is somewhat distorted due to artifacts caused by the strong laser intensity while its Raman gain is the largest among the three cases^27^. The Raman spectrum obtained in the (iii) condition of the 2nd-order diffraction DPGF scheme, on the other hand, shows the clean genuine spectral nature of cyclohexane without any unwanted artifacts.

**Figure 4:**
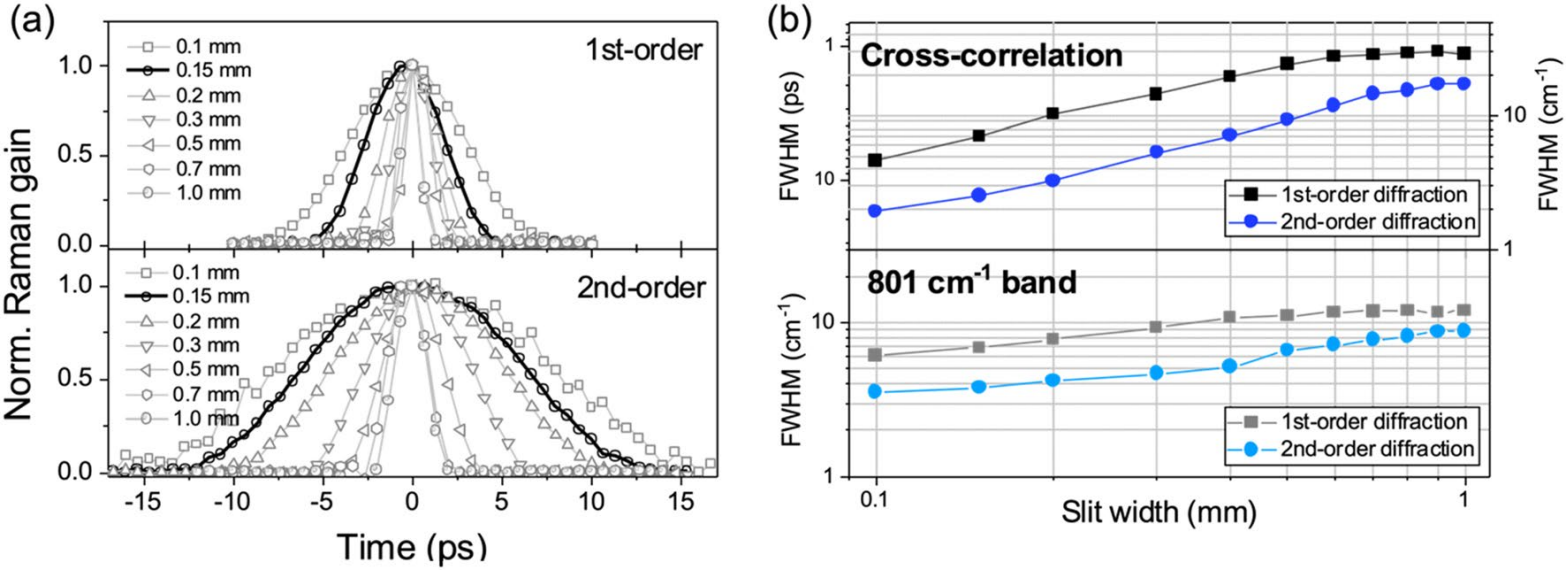
(**a**) The cross-correlation widths of stimulated Raman signal at 801 cm^−1^ of cyclohexane are shown as a function of the slit width in the DPGF setup with the 1st- (upper panel) and 2nd-order diffraction (lower panel). (**b**) FWHM values of cross-correlation signal fitted by Gaussian function are represented by using the 1st- (upper, the black squared line) and 2nd-order diffraction (upper, the blue dotted line) in temporal domain with varying the slit width. Calculated FWHM in the frequency domain by the relation of ṽ = 1/cT (c = the speed of light; T = temporal width). The experimentally measured FWHM of 801 cm^−1^ Raman band fitted by Voigt function is plotted versus the slit width with the 1st- (lower, the gray squared line) and 2nd-order (lower, the sky-blue dotted line) diffraction methods.

In Fig. 4a, the cross-correlated Raman peaks corresponding to the 801 cm^−1^ band of cyclohexane are obtained with varying the slit width in the 1st- and 2nd-order diffraction DPGF setup. The FWHM of the 801 cm^−1^ band obtained in the 1st-(2nd-) order diffraction scheme is increased from 1 (1.9) to 7.2 (17.3) ps in the temporal domain as the slit width is decreased from 1.0 to 0.1 mm, giving the expected spectral resolution of 7 (2.5) cm^−1^ at the slit width of 0.15 mm. The spectral resolution of the stimulated Raman signal depends on the delay-time between the ps Raman-pump and the fs Raman-probe pulse^11^. We have measured the spectral width of the stimulated Raman signal as a function of the Raman pump-probe delay time, showing that the FWHM becomes broadened from the change from the negative to positive delay-time, Fig. 3. It is interesting to note that the FWHM variation with the delay-time change seems to be least and monotonic at the 2nd-order diffraction condition. For the purpose of the comparison, we have chosen the optimized delay-times for the maximum Raman gains in three different conditions. The FWHM of the 801 cm^−1^ Raman band of cyclohexane decreases with deceasing the slit width, Fig. 4b, although it is found to be limited to ~ 3.5 cm^−1^ in the present setup with the 2nd-order diffraction DPGF at the slit width of 0.15 mm due to the resolution limit of the charge coupled detector employed in the current spectrometer. In order to estimate the conversion efficiency starting from the SHG output, the power loss after DPGF process has been measured as a function of the slit width (see the Supplementary Information). For the 1200 gr/mm grating (1000 nm blaze) employed here, the conversion efficiencies from the SHG output are found to be 3.3% and 1.6% from the DPGF setup in the 1st- or 2nd-order diffraction scheme at the slit width of 0.15 mm, suggesting that one could achieve the high spectral resolution of FSRS just by switching from the 1st- to 2nd-order diffraction scheme even with the cheap and easily available 1200 gr/mm grating.

**Figure 5:**
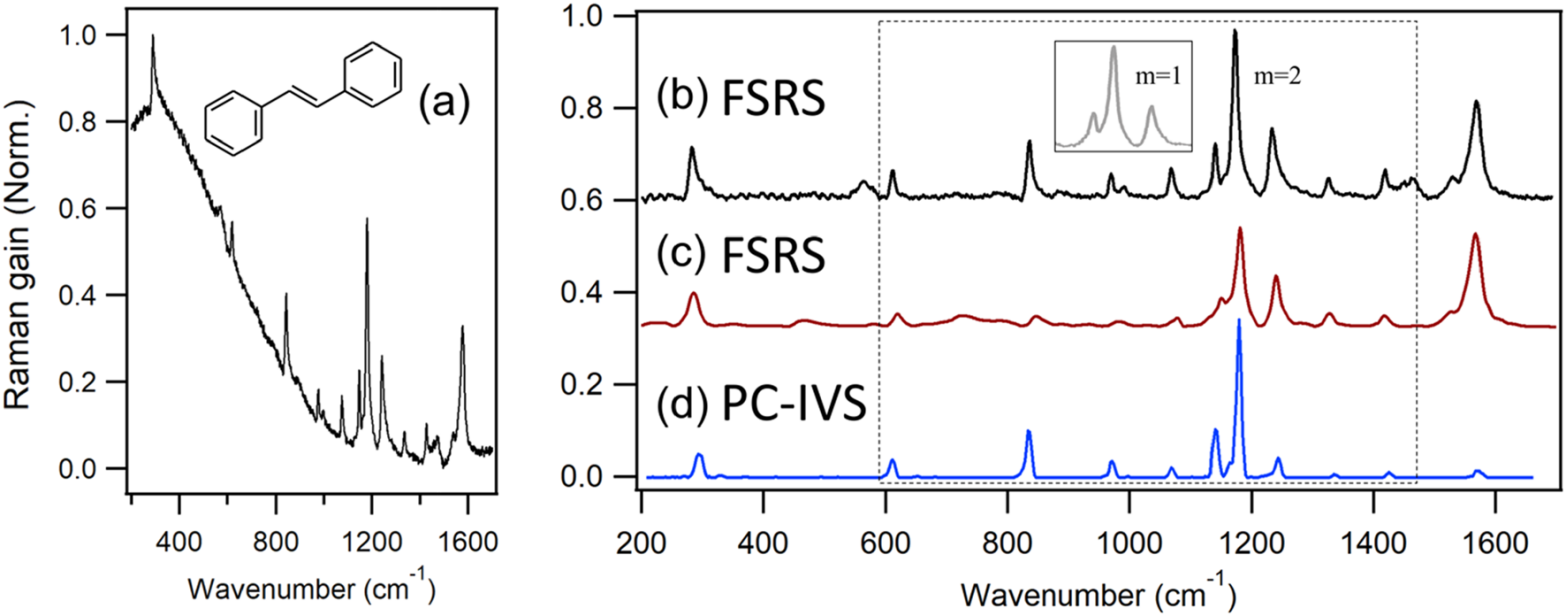
(**a**) The FSRS raw data for the exited *trans*-stilbene in *n*-hexane at the delay time of 200 fs obtained by using the 2nd-order diffraction DPGF method. (**b**) FSRS spectrum which is obtained from (**a**) after the background subtraction by the polynomial fit. Inset shows FSRS spectrum of *trans*-stilbene by using the 1st-order diffraction DPGF method. (**c**) The FSRS spectrum from Ref.^29^. Copyright 2012, American Institute of Physics. (**d**) The PC-IVS spectrum from Ref.^30^. Copyright 2014 American Chemical Society. The shoulders in (**c**) are distinctly resolved in (**b**) and (**d**).

In order to testify the spectral performance, we have obtained the FSRS spectrum of *trans*-stilbene using the Raman pump pulse from the 2nd-order diffraction DPGF. The fs actinic pump pulse at 325 nm is employed for the S_1_ ← S_0_ transition of *trans*-stilbene, while the ps Raman pump pulse at 575 nm is used for the absorption of the excited *trans*-stilbene (S_n_ ← S_1_). The sub-ps time-resolved vibrational spectrum of the excited-state then could be obtained through the stimulated Raman process by the interaction between the Raman-pump and the fs broadband Stokes Raman-probe pulse, Fig. 5a. The sharp and intense Raman bands are taken by the background subtraction from the raw data, Fig. 5b. Overall spectral pattern of the FSRS spectrum taken in this work (Fig. 5b) is quite consistent with the previously reported S_1_ resonance Raman spectra of the same molecules^28,29^. As expected, the spectral resolution of the FSRS spectrum obtained in the 2nd-order DPGF setup is certainly better than that obtained in the 1st-oder DPGF (Fig. S3). It is also interesting to note that the signal/background ratio in the raw FSR signal is much enhanced in the 2nd-order, compared to that obtained in the 1st-order DPGF (See the Supplementary Information). Dobryakov et al. had reported the high-quality FSRS spectra of the excited *trans*-stilbene using the ps Raman-pump pulse generated by the SH-BC method followed by a couple of the 1st-order grating spectral filtering processes, Fig. 5c^15,30^. The FWHM of the FSRS band was estimated to be ~ 20 cm^−1^. The stimulated Raman band obtained from the 2nd-order diffraction DPGF setup at the slit width of 0.15 mm is found to be much better resolved, giving the average FWHM value of ~ 11 cm^−1^ based on the Lorentzian function fits, Fig. 5b. Obviously, the spectral resolution of the overall FSRS spectrum taken with the 2nd-order diffraction DPGF setup is much improved from that reported in Ref.^30^. It should be noted, however, that the individual FSR bands are homogenously broadened due to the finite dephasing dynamics of associated quantum states. Our FSRS spectrum is even comparable to the Raman spectrum of the same molecule obtained by the technique of the population-controlled impulsive vibrational spectroscopy (PC-IVS) in terms of the spectral resolution, Fig. 5d^31^, although the spectral pattern from two techniques are somewhat different in terms of the relative vibrational band intensities especially above 1200 cm^−1^, of which the explanation is beyond our scope at the present time.

Many techniques for the improvement of the spectral resolution have been developed although they have pros and cons in terms of the wavelength tunability, power conversion efficiency, and the complexity of the setup. The use of the Fabry-Pérot etalon is simple and gives the excellent spectral resolution (~ 3 cm^−1^). And yet, it works only for the fixed wavelength for a particular etalon with the usually low conversion efficiency of 0.6%^1^. The SH-BC has been widely used as it provides the relatively good spectral resolution (~ 5 cm^−1^) with the high conversion efficiency of 10%. However, as the SH-BC gives rise to the fixed wavelength only, the ps-OPA should be combined for tuning the wavelength of the Raman-pump pulse. This not only makes the setup quite complicated but also broadens the spectral bandwidth and decreases the conversion efficiency. For the additional spectral narrowing, one should employ the spectral grating filter^15,30^. Compared to these methods, our FSRS setup with the 2nd-order diffraction DPGF (Fig. 1) scheme provides the very high spectral resolution of 2.5 cm^−1^ and significant conversion efficiency of ~ 2%. The simple application of the 2nd-order diffraction method in the DPGF demonstrated in this work turns out to be extremely useful for obtaining the highly resolved FSRS spectrum. In addition, the 2nd-order DPGF method is almost cost-free as there is no need for additional optical components. The implementation of our concept in the actual laboratory is extremely simple, and thus our experimental demonstration applied to get the FSRS spectrum of *trans*-stilbene here will greatly help many researchers aiming to the better FSRS spectrum in terms of the spectral resolution.

## Conclusion

In this work, we have demonstrated for the first time that the extremely high spectral resolution could be achieved in the FSRS spectrum using the DPGF technique employing the 2nd-order diffraction. The narrow stimulated Raman band has been obtained for the 801 cm^−1^ mode of cyclohexane with its FWHM of ~ 3.5 cm^−1^. The FSRS spectrum of the excited *trans*-stilbene taken by the 2nd-order diffraction is much improved in terms of the spectral resolution compared to previously reported works. The spectral bandwidth of the tailored ps Raman-pump pulse (Δν = 2.5 cm^−1^) which is attainable in the current FSRS setup is the lowest ever reported to date^1^, inspiring the more versatile application of the corresponding spectral technique.

## Supplementary Information

Supplementary Information
