# Supplementary material for: Improved spectral resolution of the femtosecond stimulated Raman spectroscopy achieved by the use of the 2nd-order diffraction method
Source: Sci Rep. 2021 Feb 9;11:3361. doi: 10.1038/s41598-021-83090-7 (PMC7873076; doi:10.1038/s41598-021-83090-7)
Supplement: Supplementary file 1 — Supplementary Information [file 41598_2021_83090_MOESM1_ESM.pdf]

Improved Spectral Resolution of the Femtosecond Stimulated Raman Spectroscopy Achieved by the use of the 2nd-order Diffraction Method: Supplemental Information

Dong-gu Kang[1], Kyung Chul Woo[1,2], Do Hyung Kang[1], Chanho Park[1] and Sang Kyu Kim[1]*

[1]Department of Chemistry, KAIST, Daejeon, 34141, Republic of Korea
[2]Current Address: Division of Chemistry and Biological Chemistry, School of Physical and Mathematical Sciences, Nanyang Technological University, 21 Nanyang Link, Singapore, 637371, Singapore
*corresponding author: sangkyukim@kaist.ac.kr

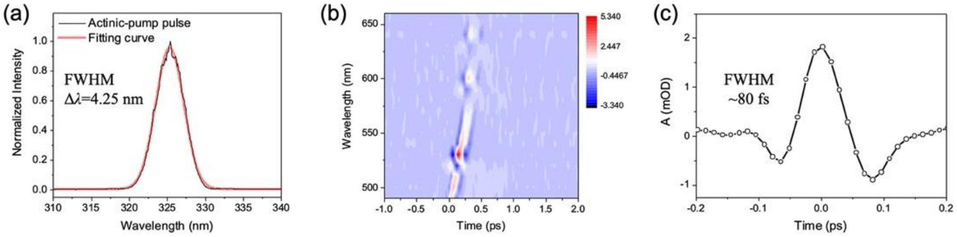

Fig. S1. The spectrum of the actinic-pump pulse centered at 325 nm is obtained by the spectrometer (Flame-S, OceanOptics) and the spectral bandwidth from the full-width-half-maximum (FWHM) fitted by the Gaussian function is 413 cm^{-1}. (b) Differential absorbance map for n-hexane solvent represents the chirp of supercontinuum Raman-probe pulse measured by cross-correlation with the actinic-pump pulse. (c) Extracted time trace of cross-correlation between the actinic-pump and Raman-probe pulses. The FWHM is obtained by the Gaussian fit.

The power conversion, P_{DPGF} through the double pass grating filter (DPGF) method from the second harmonic generation (SHG) output of ps near-infrared pulse is estimated by Equation S1:

$$P_{DPGF} = P_{SHG}(\alpha_m E_{\lambda m})^2 + Aexp(-S_{width}/\tau) \qquad \text{(S1)}$$

where, P_{SHG} is an output power of the SHG after a KDP crystal, α_m is a relative loss parameter. $E_{\lambda m}$ is a grating efficiency at multiplication of wavelength and grating order. For example, the grating efficiency at 575 nm in the 2nd-order diffraction indicates that at 1150 nm in the 1st-order diffraction. A is a fitting constant. S_{width} is a slit width. τ is an exponential decay constant which is strongly dependent on the order of the diffraction. The larger value of τ in the 2nd-order diffraction, compared to the case of

the 1st-order, indicates that the narrowing-down of the spectral resolution by reducing the slit-width is much more efficient in the 2nd-order diffraction scheme.

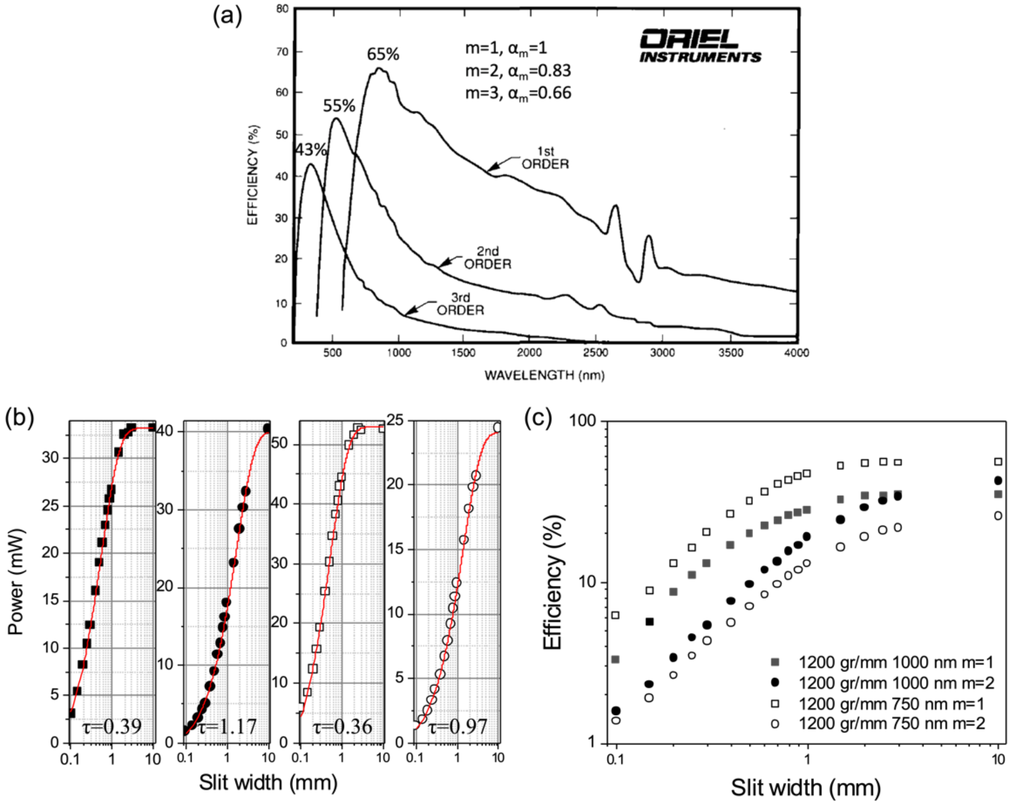

Fig. S2. (a) Typical grating efficiency curves for first, second and third orders.[1] (b) Raman-pump power after the double pass grating filter using the grating type of 1200 gr/mm 1000 nm blaze with the 1st-order (the filled square) and the 2nd-order diffraction (the filled dot) and 1200 gr/mm 750 nm blaze with the 1st-order (the opened square) and the 2nd-order diffraction (the opened dot) were measured by the power meter (FieldMaxII, Coherent). All data were fitted by exponential function and the tau-value depends on the diffraction order m. (c) Efficiencies were obtained by dividing the measured Raman-pump power by the power of the second harmonic generated pulse after the KDP crystal (~95 mW, 575 nm).

Table S1. Power conversion and efficiency after DPGF method from the SHG output using the 1200 gr/mm with two different blazed wavelength and grating order.

S_{width}=10 mm						S_{width}=0.15 mm	
Blaze (nm)	m	$E_{\lambda m}$[a] (%)	Calculated P_{DPGF} (mW)	Measured P_{DPGF} (mW)	Efficiency (%)	Measured P_{DPGF} (mW)	Efficiency (%)
1000	1	60[b]	34.2	33.2	34.9	5.4	3.3
1000	2	90[b]	54	40.4	42.5	2.2	1.6
750	1	80[c]	60.8	52.5	55.3	8.4	6.2
750	2	77[c]	39.7	24.4	25.7	1.8	1.4

[a] S-polarization
[b] Diffraction grating specification sheet of 20RG1200-1000-2 (Newport)
[c] Diffraction grating specification sheet of 20RG1200-750-1 (Newport)

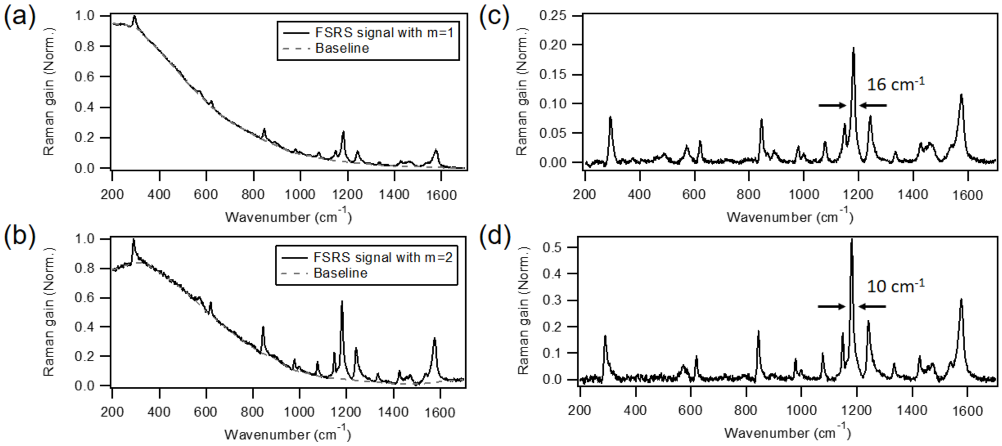

Fig. S3. Raw FSRS signals of *trans*-stilbene in n-hexane at the delay time of 200 fs are obtained by using DPGF with (a) the 1st-order and (b) the 2nd-order diffraction. After the broad background from the raw signal is subtracted by polynomial fit, each FSRS signal obtained from using (c) the 1st-order and (d) the 2nd-order diffraction methods is revealed.

References

1. Oriel Instruments Booklet of Grating Efficiency Curve
